# The Role of Power Doppler Ultrasonography as Disease Activity Marker in Rheumatoid Arthritis

**DOI:** 10.1155/2015/325909

**Published:** 2015-05-03

**Authors:** Shaloo Bhasin, Peter P. Cheung

**Affiliations:** ^1^Division of Rheumatology, National University Hospital, Singapore; ^2^Yong Loo Lin School of Medicine, National University of Singapore, Singapore

## Abstract

Structural damage in rheumatoid arthritis (RA) occurs early if inflammation is not treated promptly. Treatment targeted to reduce inflammation, in particular, that of synovial inflammation in the joints (synovitis), has been recommended as standard treat-to-target recommendations by rheumatologists. The goal is to achieve disease remission (i.e., no disease activity). Several accepted remission criteria have not always equated to the complete absence of true inflammation. Over the last decade, musculoskeletal ultrasonography has been demonstrated to detect subclinical synovitis not appreciated by routine clinical or laboratory assessments, with the Power Doppler modality allowing clinicians to more readily appreciate true inflammation. Thus, targeting therapy to Power Doppler activity may provide superior outcomes compared with treating to clinical targets alone, making it an attractive marker of disease activity in RA. However, more validation on its true benefits such as its benefits to patients in regard to patient related outcomes and issues with standardized training in acquisition and interpretation of power Doppler findings are required.

## 1. Introduction

*Concepts in Monitoring and Treatment of Rheumatoid Arthritis*. Rheumatoid arthritis (RA) is a chronic inflammatory disease associated with significant functional limitations and disability. Diagnosing RA begins with a thorough medical history of the patient, focusing on the presence, location, and duration of joint pain and stiffness as well as physical assessment of synovitis [1]. Since there is no single test to diagnose RA, clinicians use a number of tests to support the clinical diagnosis. This traditionally includes rheumatoid factor (RF), anticitrullinated peptide (anti-CCP), erythrocyte sedimentation rate (ESR), and/or serum C-reactive protein (CRP) levels as well as imaging using radiographs of the hands, wrists, and feet [2–4]. In addition, imaging with ultrasonography (US) and magnetic resonance imaging (MRI) has increased the ability to diagnose the disease earlier when the clinical presentation is unclear or when clinical synovitis is equivocal. The need to diagnose RA early and commence disease modifying antirheumatic drugs (DMARDs) has led to an updated RA classification criteria, jointly proposed by the American College of Rheumatology (ACR) and the European League Against Rheumatism (EULAR) [1].

Management of RA such as the determination of treatment decisions depends on a number of factors. Although the level of disease activity is of paramount importance, the disease duration, acknowledgement of poor prognostic factors (e.g., seropositivity for RF and or anti-CCP, erosions, and extra-articular disease), and the level of patient's disability as well as self-reported impact of disease have to be taken into account [5]. There is no single gold standard for quantifying the level of disease activity. Hence, clinicians would routinely use a number of parameters such as clinical assessment of tender and swollen joints, a global assessment of disease activity, and either an ESR or CRP level, for example, to determine the level of disease activity. A number of validated instruments for RA in the form of composite indices that combine these parameters into a score (some of them weighed) have been routinely used in clinical practice and clinical trials. This allows a standardized way to quantify the absolute level of disease activity at any given point in time. Some of these measures including disease activity score in 28 joints (DAS28), simplified disease activity index (SDAI), and clinical disease activity index (CDAI), for example, are illustrated in Table 1 [5] with their respective thresholds of levels of disease activity.

*Treating to Target to Achieve Disease Remission Is the Goal in RA*. Regular disease activity assessment with treatment adjustments according to a “treatment target” has now been universally accepted as the best practice in the management of patients with RA. The objective is to enable earlier aggressive treatment, through regular disease activity assessments and appropriate modifications of therapy, in order to achieve disease remission [6].

However, definitions of remission by clinical criteria (defined by levels of disease activity score, simplified disease activity index) do not always equate to the complete absence of inflammation. Even with more stringent criteria such as the ACR/EULAR remission criteria, a Boolean criteria that require <1 of tender and swollen joint, normal CRP, and <1 on visual analogue scale of 0–10 on patient global assessment [7] may not necessarily indicate complete absence of synovial inflammation, since subclinical synovitis can be missed by clinical assessment alone. Synovitis is frequently found by imaging, such as by US or MRI in patients considered to be in remission, and is associated with adverse clinical and functional outcomes [8]. Some have argued that targeting therapy to PDUS activity may provide better outcomes compared with targeting therapy to clinical targets alone [9]. This makes it an attractive and feasible marker of disease activity in RA.

**Figure 1:**
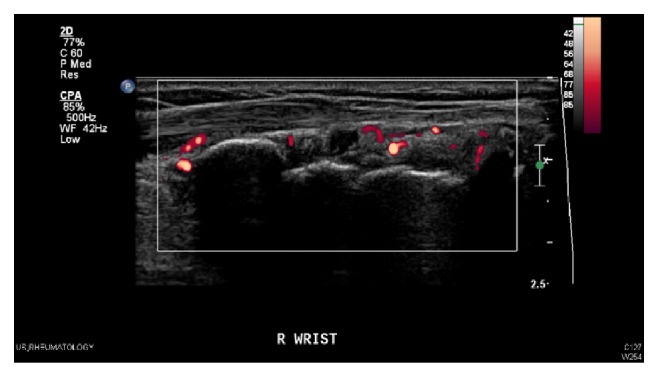
Wrist joint with Power Doppler synovitis in patient with RA.

*Ultrasonography in Rheumatoid Arthritis*. Within the last decade, musculoskeletal ultrasonography has played an increasingly important role in the evaluation and monitoring of patients with chronic inflammatory arthritis. US can readily evaluate synovitis, a pathological hallmark of RA at both the anatomic and vascular levels. There are 2 US techniques that are of use:

- B-mode or gray scale US: imaging of anatomic structures, which enables visualization of synovial hypertrophy and/or effusion,
- Power Doppler US (PDUS): blood flow detection, which allows visualization of the movement of blood vessels, therefore detecting increased microvascular blood flow seen in active synovitis (Figure 1).

*Power Doppler Ultrasonography*. PDUS is based on Doppler effect, which consists of the change of frequency of a sound beam reflected back to the source when it encounters a moving object. Doppler technique detects the movement of red blood cells in vessels. PDUS technique is more accurate than conventional Color Doppler. Color Doppler encodes direction and velocity of blood flow while PDUS displays the total integrated Doppler power in color therefore increasing the sensitivity to detect strength of flow from small vessels and low velocity flow which is usually the case in RA patients [10].

*PDUS Grading*. The most frequently applied method of grading severity is by a semiquantitative scoring system in which the intensity of the synovial blood flow is graded in a four-step scale [11] in Table 2.

This semiquantitative grading system is considered a practical way to standardize PDUS measurement in RA. Compared to other more sophisticated ways of quantifying flow (computer-assisted measurement of color pixels, resistance index, and analysis of Doppler curves, contrast-enhanced Doppler US) [12], it does not involve contrast media or further computer-assisted evaluation software. In addition, it is validated in the diagnostic and therapeutic outcome evaluation of patients with RA in various settings.

*PDUS Settings*. Although using the PDUS setting is usually preferred, in newer high-end machines, Color Doppler may have better sensitivity than PDUS [13, 14]. In low and intermediate range machines, PDUS always has the highest sensitivity. Therefore, ensuring good standardization of settings for the machine is important usually by a technician from the manufacture.

The pulsed repetition frequency (PRF) is Doppler sampling frequency of the transducer (how many pulses are emitted per second) and is reported in Hz. This is important in RA as the goal is to detect as much flow as possible often in the small joints such as metacarpal phalangeal (MCP), proximal interphalangeal (PIP), and metatarsal phalangeal (MTP) joints. The settings should therefore be able to achieve the highest sensitivity without noise artifacts [15]. The usual recommended settings would be adjusting to the lowest possible PRF, wall filter, and persistence. Gain on the threshold to noise should be achieved with the focus placed on where the highest sensitivity is required with all priority to color [15]. Adjusting and optimizing this correctly will have great impact on the ability to see inflammatory flow. Adjusting many parameters of the PDUS is not done at every examination. Fortunately, there is little difference usually from patient to patient and from joint to joint with respect to PDUS settings. An exception is the hip joint because of its deep location. Once the sensitivity of the PDUS has been optimized, the settings may be saved as a set-up, which the machine reverts to at every new exam [15].

Using the same set-up with the same machine is recommended to compare treatment response longitudinally or between patients. The effect of using different machines, PDUS modalities, and settings has a considerable influence on the quantification of inflammation in RA patients and this should be taken into account in multicenter studies [16]. Much of the variation in the literature concerning detection between hyperemia and normal flow may be attributed to differences in machine and settings. To overcome these drawbacks, experts in this field have collaborated to standardize scanning methods, define abnormalities, determine reliability, and promote education [17–27]. Increasing number of training courses is available for rheumatologists to learn how to use PDUS in their clinical practice [28–34] with a huge growth in the uptake of US usage over the last 5 years especially in Europe [35].

## 2. The Role of PDUS in Rheumatoid Arthritis

For a standard measure of disease marker to be endorsed as a valid outcome measure, the measurement should fulfill a number of metrological properties. In rheumatology, the Outcome Measures in Rheumatology (OMERACT) had developed and recommended some of these principles, due to the lack of standardized valid outcome measures in rheumatic diseases. The OMERACT filter has recommended an outcome measure should be [36]

- truthful (construct, content, and criterion validity),
- feasible,
- discriminatory (sensitive to change and reliable).

Therefore, for PDUS synovitis to be a valid outcome measure in disease activity assessment in RA, it should fulfill these properties as well.

### 2.1. PDUS versus Clinical Assessment of Synovitis

The link between synovial vascularity and ultimate joint damage makes the differentiation between inactive and actively inflamed synovium in the rheumatoid joint an important issue in management of patients with RA [37, 38]. It is well known that traditional clinical signs such as the tender and swollen joint count and composite scores of disease activity that includes just clinical measures do not entirely reflect active inflammation as detected by PDUS [39]. Tender joint counts do not correlate with ultrasound-detected joint effusion, synovitis, or PDUS signal, in contrast with the swollen joint counts, for example [40]. In addition, US detected subclinical synovitis is not well appreciated by clinical assessment alone. Wakefield et al. [41] reported in early oligoarthritis that the proportion of patients with US-determined synovial hypertrophy in a “painful only” group was much lower (33%) than that in a clinically determined synovitis group (79%). US also has the added advantage of being able to differentiate whether the joint is actively inflamed or not by PDUS [41–44].

Other studies comparing clinical and US assessment have reported a stronger correlation between US and physical examination of joint swelling than joint tenderness [45, 46]. Given the close correlation of PDUS data with both histological and MRI assessments of synovial inflammation [47–49] and the ability of PDUS to detect increased blood flow, it may be a suitable bedside tool in routine assessment of synovitis. Both quantitative and semiquantitative PDUS scores have the possibility to grade the disease activity in comparison with the standardized joint count, which usually allows assessment of presence or absence of swelling or pain only.

### 2.2. PDUS versus Laboratory Markers of Inflammation

The concurrent validity of PDUS is supported by its significant correlations with CRP or ESR, which are laboratory markers included in several validated composite disease activity in RA [50]. Kawashiri et al. found that PDUS scores not only correlated with composite disease activity indices, but also positively correlated with serum biomarkers such as MMP-3, VEGF, and tissue inhibitor of metalloproteinases-1 (TIMP-1). MMP-9 is important for the budding of endothelial cells, and TIMP-1 is an inhibitor of MMP-9; both are elevated in serum and synovial tissues of patients with RA. Since the budding of endothelial cells is an early step in angiogenesis, MMP-9 may be important in the early phase in the development of synovitis in RA [51]. Others have demonstrated PDUS correlated significantly with serum levels of IL-6 and VEGF in patients with early inflammatory arthritis which are further implicated in the pathogenesis of the PDUS signal from inflamed synovial joints [52].

The correlation between PDUS and DAS28 is not surprising once the correlations to swollen joint count and CRP were established; both are part of DAS28. Thus, PDUS could perhaps be developed to supplement the joint counts in DAS28. Excellent correlations between DAS28 calculated with clinical swollen joint count and swollen joint count derived by PDUS have been demonstrated [53]. As an outcome measure, US including PDUS is at least as relevant as physical examination but further studies are required to achieve optimal scoring system [54].

### 2.3. PDUS versus Histopathology

Walther et al. were first to correlate PDUS findings with synovial histopathology, supporting the value of PDUS. The correlation between the quantitative results of PDUS and the pathologists' estimation of vascularity was excellent (*r* = 0.89, *p* < 0.01) [55]. The best correlation was found when a semiquantitative 4-point grading scale was used by both the sonographer in assessing PDUS signals and the pathologist in assessing the degree of vascularity by histopathology. Thus, PDUS provides a reliable and accurate method for visualizing blood flow in the synovial tissue [55]. Furthermore, Motomura et al. replicated this and demonstrated significant correlations between PDUS and histopathological findings in RA patients with active synovitis (*r* = 0.54, *p* < 0.01) [56].

### 2.4. PDUS versus MRI

Although direct comparison of PDUS and MRI is difficult [57], PDUS synovitis appears to correlate with synovitis detected by MRI [44, 58]. With T1-weighed MRI as the reference standard, PDUS had a sensitivity of 0.70 and specificity of 0.78 for detecting inflammation in the small joints of the hands [43]. Although US is not as sensitive to detect bone erosions than MRI, US detected erosions have a high specificity [43]. In addition, PDUS synovitis may be able to predict future erosion progression in the joint even though it is unable to detect bone marrow abnormalities that MRI is capable of. However, gadolinium contrast administration is still required for the assessment of synovitis or tenosynovitis in RA by MRI.

Like MRI synovitis grading, appropriate training is also required for assessment of PDUS synovitis.

### 2.5. Monitoring of Disease Activity in Rheumatoid Arthritis Using PDUS

There is an increase in use of PDUS for monitoring joint inflammatory activity in patients with RA [59–61]. This includes routine clinical monitoring with good correlations of change in DAS28 with the change in PDUS score [62].

The potential role of PDUS in the follow-up of RA patients has been demonstrated more than 10 years ago [63]. A good correlation between the clinical response to infliximab and decrease in synovial thickness and PDUS signal was demonstrated, indicating that PDUS could be a feasible and sensitive tool to measure the response to therapy [63]. Subsequently, other groups have demonstrated similar findings that PDUS activity reduces significantly with treatment by other anti-TNF agents [53, 64]. This was also confirmed by another study in RA patients treated with corticosteroids [65] as well as intra-articular steroids [66]. This is important especially in terms of monitoring for response in treatment.

Decrease in PDUS synovitis can be seen as early as 2 weeks with treatment [66, 67]. Improvements in PDUS synovitis are at least as sensitive as changes in clinical and laboratory indices of disease activity [68]. When swollen joint count in the DAS28 was replaced with that derived by PDUS, changes in US derived DAS28 were consistent with and significantly correlated with changes in the original DAS28 [53]. These results demonstrate the validity of PDUS in longitudinal assessment and monitoring of disease activity in RA.

### 2.6. The Role of PDUS Activity in Prognosis in RA

There is evidence that RA patients continue to have radiographic progression despite achieving clinical remission [69–71], which indicates the inadequate sensitivity of conventional approaches in detecting active synovitis and predicting structural damage. PDUS synovitis better reflects pathologic alterations of rheumatoid synovial inflammation in patients than that by gray-scale or clinical synovitis assessment [59, 72–74].

*(i) Patients with Early Rheumatoid Arthritis*. Residual PDUS synovitis is predictive of clinical flare-ups in patients with early RA (median disease duration of 4 months) treated by conventional DMARDs [74]. Although the predictive validity of PDUS synovitis for structural damage has not been well described in patients with early RA, Kawashiri et al. had demonstrated patients with early RA with PDUS subclinical synovitis were associated with more bone erosions [75].

*(ii) Patients with Established RA*. In patients with established RA, the qualitative importance of subclinical synovitis was first described by Brown et al. [72], by showing that joints with PDUS signals may continue to have structural deterioration irrespective of the achievement of good clinical status. PDUS synovitis may also be present in long-standing established RA patients even after achieving clinical remission [76].

*(iii) Patients on Conventional DMARDs*. Saleem et al. showed that RA patients in clinical remission with residual PDUS synovitis would develop clinical flare-ups during treatment with conventional DMARDs [77]. These data strongly suggest that RA patients in clinical remission with residual PDUS synovitis do not achieve “true” remission and are at risk for subsequent disease flare. In addition, Naredo et al. reported a positive relationship between PDUS synovitis and subsequent radiographic progression in patients treated with DMARDs [40, 78].

*(iv) Patients on Biological DMARDs*. PDUS synovitis has been demonstrated to be a useful tool in monitoring patients under biologic DMARDs, and its predictive ability for radiographic progression has been validated [53, 79–82]. In addition, Hama et al. showed that total PDUS scores were a strong predictor for radiographic progression in RA patients receiving tocilizumab [83]. PDUS may also assist with the determination of retreatment with rituximab, guided by the presence of PDUS synovitis that is present before clinical signs are present [84].

### 2.7. Reliability

US, in particular, PDUS, has been known to be operator dependent and, therefore, like clinical assessment of synovitis, is liable to interobserver variation. A systematic review on 35 studies demonstrated that US reliability was good in still-image interpretation (both intraobserver and interobserver), particularly with PDUS mode, especially by experienced ultrasonographers. However, PDUS image acquisition was less reliable. Reliability in semiquantitative and binary scoring appeared similar, and the knee was the most reliably assessed joint, including image acquisition. The small joints of the hands, which are the most studied in US reliability studies, had good reliability results in still-image interpretation, but image acquisition was variable. Results in the feet were poor and understudied [85]. Recently, Hammer et al. proposed a comprehensive approach to improve synovitis scoring, including PDUS which includes training sessions to achieve consensus on scoring as well as incorporating the use of reference atlas of representative images of each score for all examined joints. This study demonstrated excellent reliability for grey scale and PDUS scoring of a large number of joints in RA patients [86].

## 3. Limitations

The lack of standardization of US examination method previously and settings for PDUS can limit the use of this technique in clinical practice. Although it is generally accepted to use a semiquantitative scoring system, it is not the gold standard. PDUS is operator dependent and liable to reliability problems. It is extremely sensitive to tissue movement, especially at low PRF, which can result in flash artifacts [87]. Without strict standardization to determine what is normal and abnormal, interobserver reliability especially in acquisition and image interpretation is still a concern [85]. Studies have attempted to address the question of reliability; however, data appear conflicting [88–90]. Normal blood flow in the synovium may lead to the presence of hyperaemia being overinterpreted in machines with a very sensitive PDUS setting [13]. Even when using the same machine, different examiners may obtain very different results depending on how the PDUS is adjusted, scanning technique, or the presence of artifacts [15]. Reliability, particularly for acquisition, can have the potential to improve with standardized teaching programs, development of consensus guidelines, and improvement in machine quality [19, 27]. Another important factor determining reliability is the experience of the ultrasonographer. In studies where observers had limited knowledge of US, improvement in acquisition reliability after standardization and training was noted [91].

Feasibility can also be an issue. With the prices of obtaining an adequate machine reducing, accessibility is improving. However, it is not feasible to scan all the joints in the body, as it is time consuming. There is still controversy about the optimum number of joints to scan for diagnosis, monitoring of disease, and treatment response as well as assessment of disease remission. A recent review of studies evaluating the use of US in RA had included the wrist and MCP joints of the dominant hand, usually in the dorsal position as the minimum number of joints to scan [92, 93]. Based on the results of this review, it seemed that it is not necessary to scan large joints when diagnosing RA or evaluating disease remission. In general, the more the joints that are scanned, the higher the chance of finding PDUS signs [92]. However, it would be problematic to only scan the small joints of patients in cohorts where RA presents with predominant residual large joint synovitis [94]. A new abbreviated 7-joint ultrasound (US7) score had been proposed which combines soft tissue (synovitis and tenosynovitis/paratenonitis) and destructive lesions (erosions) in a composite scoring system for use in the monitoring of disease activity in RA [95]. This is potentially a feasible way to monitor for disease activity although further validation in other cohorts is required.

Another limitation is that although PDUS is prognostic of disease and radiographic outcomes, we have limited data on its relationship with patient reported outcomes. Since PDUS as a disease marker is considered to be more accurate and sensitive, the longitudinal impact of treating to target according to PDUS synovitis and its subsequent effects on patient reported outcomes are lacking. In addition, we do not know what degree of residual PDUS activity is acceptable for predicting disease outcomes, such as remission or relapse or even for clinically important radiographic progression. Currently, there is a multicenter randomized controlled study evaluating this question using ultrasound as the treatment target for RA [9].

## 4. Conclusion

PDUS correlates significantly with clinical findings and common and novel inflammatory markers along with synovial histopathology in patients with RA. It has the ability to detect subclinical synovitis not appreciated by clinical examination alone. Studies have shown that PDUS is valid, reliable, sensitive to change, and largely feasible. Therefore, it has a potential role in standard monitoring and follow-up of patients for response to treatment as well as prediction of future structural damage.

However, there are certain limitations including the lack of standardization of PDUS scoring and settings, leading to a high level of inter- and intraobserver reliability, controversy in the number of joints to be assessed for diagnosis, and monitoring of disease as well as its clinical implications relating to patient reported outcomes. These limitations need to be fully addressed before PDUS can be considered as a universally accepted marker of disease activity in RA.

## Figures and Tables

**Table 1:** Composite indices measuring disease activity in rheumatoid arthritis.

Instrument	Components	Thresholds of disease
		Activity levels
Clinical disease activity index (CDAI) (range from 0 to 76.0)	Tender joint count	Remission: ≤2.8
	Swollen joint count	Low activity: from >2.8 to 10.0
	Physician global assessment	Moderate: from >10 to 22.0
	Patient global assessment	High: >22.0

Disease activity score in 28 joints (DAS28) (range from 0 to 9.4)	Tender joint count	Remission: <2.6
	Swollen joint count	Low activity: from ≥2.6 to <3.2
	Patient global assessment	Moderate: from ≥3.2 to ≤5.1
	ESR	High: >5.1

Simplified disease activity index (SDAI) (range 0 to 86.0)	Tender joint count	Remission: ≤3.3
	Swollen joint count	Low activity: from >3.3 to ≤11.0
	Patient global assessment	Moderate: from >11.0 to ≤26.0
	CRP	High: >26.0

**Table 2:** Semiquantitative grading of severity of Power Doppler signal in rheumatoid arthritis.

PDUS grading [11]	
Grade 0: being with no signal visualized	
Grade 1: having one single or several vessels visualized	
Grade 2: less than 50% of the region of interest having signal	
Grade 3: being more than 50% of the region of interest having signal	
